# A large-scale examination of the effectiveness of anonymous marking in reducing group performance differences in higher education assessment

**DOI:** 10.1371/journal.pone.0182711

**Published:** 2017-08-15

**Authors:** Daniel P. Hinton, Helen Higson

**Affiliations:** Aston Business School, Aston University, Birmingham, United Kingdom; University of Guelph, CANADA

## Abstract

The present research aims to more fully explore the issues of performance differences in higher education assessment, particularly in the context of a common measure taken to address them. The rationale for the study is that, while performance differences in written examinations are relatively well researched, few studies have examined the efficacy of anonymous marking in reducing these performance differences, particularly in modern student populations. By examining a large archive (N = 30674) of assessment data spanning a twelve-year period, the relationship between assessment marks and factors such as ethnic group, gender and socio-environmental background was investigated. In particular, analysis focused on the impact that the implementation of anonymous marking for assessment of written examinations and coursework has had on the magnitude of mean score differences between demographic groups of students. While group differences were found to be pervasive in higher education assessment, these differences were observed to be relatively small in practical terms. Further, it appears that the introduction of anonymous marking has had a negligible effect in reducing them. The implications of these results are discussed, focusing on two issues, firstly a defence of examinations as a fair and legitimate form of assessment in Higher Education, and, secondly, a call for the re-examination of the efficacy of anonymous marking in reducing group performance differences.

## Introduction

Within Higher Education, there are three main types of summative assessment used to assess students on an individual basis: written examinations, coursework, and oral examinations. For the purposes of clarity in this paper, these grading constructs are defined in the following ways, in line with convention in UK Higher Education: Written examinations are timed tests of a student’s knowledge and understanding of the course material, administered in a controlled environment, in which an invigilator is present to ensure students adhere to procedure. They examine students by having them respond to essay questions or having them provide a series of written short answers to less complex questions. They typically do not allow students to refer to reference material (‘closed book’ examinations), but, occasionally, will allow students to bring material such as text books to use to aid them (‘open book’ examinations). Coursework assignments are written pieces of work that are typically set weeks or even months ahead of the submission deadline. They set the student a problem or essay question, to which they must form an answer that is much longer than those in written examinations, typically between 1000 and 4000 words. The student crafts their answer to the question posed over a relatively long period of time, supporting their answer with reference to the source material they have available. Oral examinations are assessments in which the student demonstrates their knowledge and understanding to their assessors verbally. They may be required to make a short presentation, to role play a real-world scenario, or be asked a series of questions, similar to a viva voce examination.

Written examinations are the most common method used to assess students in Higher Education. Proponents of the use of written examinations to assess students frequently claim that they are superior to assessment by coursework as they reduce the scope for academic misconduct such as plagiarism and collusion, given the strictly controlled nature of the environment in which they are administered [1]. This need for control of academic misconduct is one that is of growing importance in the digital age. Massively increased access to information via the Internet has brought with it a growing temptation for students to resort to copying and pasting electronic sources into their coursework without citation [2].

However, in spite of these benefits over coursework assessments, it has been demonstrated many times in the past that performance in written examinations–all else being equal–tends to favor certain social groups over others. Kysel [3] observed very large disparities in the performance of many ethnic minority student groups in written examinations. Furthermore, it has been observed that, while academic performance of some ethnic groups has improved over time, there are some for whom no such improvement has been observed, suggesting a gradual widening of performance differences between these ethnic groups over time [4].

Demographic group differences in written examination performance are not limited to those observed between students of different ethnic groups. Stobart, Elwood and Quinlan [5] observed that in most subjects, with the exception of mathematics and some of the natural sciences, female students outperform their male peers. It has been observed that these patterns of difference become more pronounced as students progress through their school career [6].

It would appear that performance differentials also exist between students on the basis of socio-environmental factors. Sammons [6] additionally noted similar patterns of performance differences between students from low- and high-socioeconomic status backgrounds as she did for differences between males and females. Furthermore, it has been argued that many of the performance differences observed between students of different ethnicities is attributable to group-level differences in socio-economic factors, particularly the type of school attended [7].

### Previous attempts to address bias in assessment

Pedagogic practitioners are well aware of these performance disparities, and have tended to account for them in terms of a combination of both conscious and unconscious biases to which assessors of students’ work are subject. It has been extensively demonstrated that a number of specific biases (characterized as being based on evolutionary cognitive shortcuts) can influence their perception–and, by extension, their assessment–of a student and their work [8]. The most relevant of these when discussing bias in academic assessment is the Similarity Effect. The Similarity Effect is the phenomenon that assessors tend to rate those that they perceive to be similar to them more favorably than those that they perceive to be dissimilar to them, most frequently in terms of gender or ethnicity. It has been observed that the Similarity Effect, when combined with other, similar idiosyncratic rating tendencies, can account for up to 62% of the variance in assessments of performance [9].

In response to criticisms levelled at the use of written examinations on the basis of these previously observed patterns of performance difference, some Higher Education institutions have implemented anonymous marking, the rationale being that assessors will be less prone to both conscious and unconscious biases when marking anonymous scripts [10]. There is some evidence to suggest that these interventions have been at least partially effective in reducing bias when compared to non-anonymous marking [11]. However, this approach overlooks the possible role that seemingly unrelated demographic factors such as ethnicity, gender and socio-economic status could have on assessment *performance*, as opposed to how assessments are marked.

### Accounting for performance differences by mode of assessment

Sitting a written or oral examination is a stressful event for a student to have to endure. It has been consistently demonstrated that the prospect–and experience–of a written or oral examination can frequently lead to acute stress in students [12].

Our current understanding of how stress affects performance is derived from the work of Selye [13], Lazarus [14], and those who have built upon their work. It is now accepted that anxiety and performance–particularly on complex tasks–are related to one another according to a Yerkes-Dodson (inverse U-shaped) curve [15]. If someone is under a great deal of pressure, their performance will suffer markedly as the degree of pressure increases (a state that Lazarus termed ‘*distress*’). However, when a person experiences low-moderate levels of stress, it can have a motivating and performance-enhancing effect (termed ‘*eustress*’).

In contrast to written or oral examinations, which can cause acute stress, coursework assignments may cause lower levels of stress but over a more protracted period [12]. While potentially detrimental to the performance of a few students who are particularly prone to stress, it is likely that assessment by coursework causes distress states in fewer students, and is likely to cause beneficial eustress states in more students–and for longer periods of time–than do examinations. Therefore, it stands to reason that coursework should give more students the opportunity to perform to the best of their ability than written or oral examinations do, but this alone does not account for why certain social groups appear to perform substantially less well than others in examinations at the group level.

One possible explanation that could account for these disparities in group performance in written and oral examinations is Stereotype Threat. It has been shown that female candidates and those from certain ethnic minorities can experience a phenomenon by which their performance on timed tests of cognitive ability is adversely affected due to anxiety about the test and resultant poor performance on the first few questions of the test [16]. It is not unreasonable to extrapolate that this effect could also exist for certain candidates in the pressured environments within which both written and oral examinations take place, though is unlikely to manifest during individual coursework assessment.

### The present research

The present research aims to address these specific knowledge gaps in the literature and to clarify, most importantly, whether anonymous marking has been as effective as its proponents claim it to have been in reducing bias in Higher Education assessment. If it transpires, however, that group differences still exist in coursework and written examination marks and there has not been a significant reduction in between-group mean assessment score differences, it would potentially cast doubt on the efficacy of anonymous marking in eliminating bias in these forms of assessment.

Furthermore, comparison is made between mean differences in written examination marks between different social groups and those in coursework marks since the implementation of anonymous marking. If the differences in mean score between social groups are more pronounced for written examinations than they are for coursework marks, the implications for pedagogic practice may be that written examinations are systematically disadvantaging certain student groups in assessment throughout their university careers, in spite of the anonymous marking initiative to combat this. If this were the case, the argument for the continued use of written examinations as a fair mode of assessment in Higher Education practice would be difficult to sustain.

To this end, archival data will be analyzed from a Higher Education institution in the United Kingdom for which issues of fairness are particularly salient. The institution prides itself on having an extremely ethnically and socio-economically diverse student body, one that is much more diverse in these respects than is typical of Higher Education institutions, particularly those in the UK. It can be argued, therefore, that it is of paramount importance that assessment practices at this university can be judged to be fair to all social groups. Given the growing diversity of student populations, the issue of fairness in HE assessment is one that requires urgent attention.

In the specific case of performance differences across levels of socio-economic status, a conceptual problem presents itself in the form of Range Restriction. Range Restriction is a phenomenon most frequently cited in the occupational assessment literature, in particular that which examines the predictive validity of psychometric tests in job selection. The key issue is that, in a study of predictive validity, a reliable criterion of future performance is needed, one that, ideally, considers the full range of levels of future performance.

The problem of Range Restriction is one that is frequently overlooked in the educational assessment literature dealing with performance differences between socio-economic levels. Studies of this kind tend to conceptualize socio-economic status as a dichotomy comprising groups of students who have previously attended private educational institutions, and those who have attended state-funded ones [17]. However, private institutions are–almost without exception–selective to some degree in the pupils that they accept. By contrast, the vast majority of state-funded institutions are non-selective, basing acceptance of pupils on other criteria such as geographical location of residence. The more selective nature of private schools means that poorest performing students in the wider population would have not met the eligibility criteria for acceptance to these schools, so the range of levels of performance within them must necessarily be restricted when compared to that of state schools, for which no such criteria apply. The implication of this is that many of the previous observations in the literature that students who attended private schools out perform their state-schooled peers in terms of academic success–often attributed to higher quality of education within private institutions–may be, at least in part, attributable to Range Restriction. The effect of this is that Range Restriction may exaggerate academic performance differences between socio-economic groups when conceptualized in this way.

In investigating the impact of socio-economic status (SES) on assessment scores, a novel approach to operationalizing SES as a variable will be implemented. Due to the problem of Range Restriction, it is argued that using school type as a proxy for SES is unreliable, leading, potentially, to overestimation of between-group mean differences. To combat this, a measure of Social Status (a proxy for SES) proposed by Barratt [18] will be used. The Barratt Simplified Measure of Social Status (BSMSS) is a short questionnaire, based upon the seminal work of Hollingshead [19]. It estimates SES on the basis of a respondent’s educational level and occupational prestige, taking into account those of their mother and father. The prestige of an occupation is classified as belonging to one of nine points on a hierarchy, classifications being derived from a large sample of US people’s perceptions of relative prestige. While far from a perfect measure of socio-economic status (a construct that considers additional factors such as accrued wealth), it is expected that this proxy measure of SES will not suffer the same problem of Range Restriction as classic conceptualizations have done.

Finally, the focus of the majority of the research literature to date in the area of performance differences in educational assessment has been on differences between groups at the secondary education level. Comparatively little research has been published that investigates the existence of performance in tertiary educational settings. The present research represents a ‘state of the art’ analysis of one of the key issues currently facing pedagogic practitioners.

#### Hypotheses

In investigating the nature of performance differences in Higher Education assessment, the present study proposes the following hypotheses:

H_1_: Group differences on written examinations and coursework between males and females, and between students from different ethnic groups will be less pronounced post-implementation of anonymous marking than pre-implementation.

Since its implementation at the start of the 2005/2006 academic year, anonymous marking has been implemented for all assessment by way of written examination and coursework at the institution on which the present study focuses. Due to this anonymization of students’ work, it is expected that the variance in scores on these assessments that can be attributed to idiosyncratic rating tendencies (particularly the Similarity Effect) will have been eliminated in these kinds of assessments as it will have become impossible for assessors to identify a student’s gender or ethnic background on the basis of their name. By contrast, this anonymization is impossible for oral examinations for fairly obvious reasons, so it is expected that group differences in assessment score will not have changed substantially since the implementation of anonymous marking.

H_2_: Due to the more stressful nature of these types of assessment, the magnitude of differences between males and females, and between ethnic groups will be larger for assessment by both written and oral examination than they will be for coursework assessment.

The high-pressured environment of written and oral examinations is likely to be a stressor for most–if not all–students, but it is also possible that additional stressors will manifest for certain social groups in the form of Stereotype Threat. These additional stressors may cause these students to move from a state of eustress to one of distress, negatively impacting on their performance. By contrast, in coursework assessment there is no such pressure (or, at the very least, it is much reduced when compared to both forms of assessment), potentially allowing more students to function in a state of eustress, with little or no detriment to performance.

H_3_: Assessment performance differences between students from high-SES and low-SES backgrounds will be less pronounced when SES is represented as a function of highest educational level and parental occupational level than when it is represented by type of school attended.

The range of levels of student ability within selective schools will necessarily have been restricted. This Range Restriction means that students from these schools will necessarily demonstrate higher mean assessment grades across the board when compared with their peers from other, non-selective institutions (the poorest performing applicants for these schools having already been selected out). When SES is represented in a more objective way–such as through use of the BSMSS [18]–it is likely that group differences between high- and low-SES students will be much reduced.

## Method

### Study overview

A set of archives containing data from previous students (N = 31710) were obtained from a Higher Education institution in the United Kingdom. Data were obtained for students leaving the university over a twelve-year period, between the 2000–2001 and 2012–2013 academic years (inclusive). The archives recorded demographic data for these students as well as data for every summative assessment they took as part of the degree course they pursued while attending the university. These data archives were consolidated into a single data set (S1 File) that was then recoded to allow statistical analyses to be conducted.

### Participants

Data within the archives pertains to 31710 previous students at a Higher Education institution in the United Kingdom. Of these students, 16212 were male (51%) and 15497 were female (49%). How students categorized their ethnicity was broken down as follows: 23.8% classified themselves as being White–British, 0.5% as White–Irish, 16.6% as White–Any other White Background, 18.6% as Asian or Asian British–Indian, 12.6% as Asian or Asian British–Chinese, 1.5% as Asian or Asian British–Bangladeshi, 7.4% as Asian or Asian British–Pakistani, 6.1% as Asian or Asian British–Any other Asian Background, 4.5% as Black or Black British–African, 1.1% as Black or Black British–Caribbean, .6% as Black or Black British–Any other Black background, .8% as Mixed–White and Asian, 0.4% as Mixed–White and Black Caribbean, 0.3% as Mixed–White and Black African, 0.7% as Mixed–Any other Mixed background, and 1.9% as Other ethnic group (not specified). Ethnicity data was unavailable for 2.6% of the total sample.

### Procedure

In addition to the demographic data recorded for each participant, data was recorded for each summative assessment that student took while studying for their degree. As a matter of course, the university’s Registry department holds records of assessment scores for each individual component of a module that contributes to a student’s overall module grade. For each of these components, the dataset reported the score obtained by the student as a percentage and the type of assessment. The archive additionally reported the year of entry to the university, year of leaving, each student’s qualifications obtained prior to joining the university, the type of secondary or further education institution they attended, and the occupation of their highest earning parent or guardian.

Data were recoded in Microsoft Excel 2010 then imported for analysis into SPSS version 20. Prior to importation, data were first cleansed to eliminate cases of missing data for critical variables. For each student, a mean written examination, coursework and oral examination mark was calculated as a percentage. The dataset was then cleansed to ensure that each student represented within the dataset had a mean assessment grade in at least one of these assessment categories. Post data cleansing, 30674 students remained in the dataset.

Data within the dataset were then recoded to allow for robust analyses. To simplify analyses by ethnicity, and because there were relatively few cases in many of the ethnic classifications within the dataset, it was judged that ethnicity should be conflated to a new, simplified variable of White, Black and Asian students to allow for more robust comparisons between these groups. As ethnicity is a hugely difficult construct to represent, and due, again, to relatively small samples sizes in many classifications, it was judged appropriate in this instance to exclude those participants who reported themselves to be of mixed ethnicity from the analyses of ethnic group differences. This new variable was then dummy coded into three new variables, each representing one of the ethnic categories in the simplified variables, allowing them to be analyzed in a linear regression model.

The type of educational institution that each participant attended prior to university was coded as an ordinal variable based on the typically observed patterns of achievement of each type of institution relative to one another. On the basis of records collected at the national level of mean A-level achievement between 2003 and 2012 by Cambridge Assessment using data obtained from Inter-Awarding Body Statistics [20], independent schools were coded as the highest achieving, followed by grammar schools, sixth form colleges, comprehensive schools and Further Education colleges.

A new variable to represent each student’s approximate level of Socio-economic Status was then calculated based on the method recommended by Barratt [18]. Student qualifications prior to joining the university were coded ordinally on the basis of Barratt’s 7-point scale, equivalences of UK qualifications being calculated using recommendations published by the Office of Qualifications and Examinations Regulation [21] and equivalences of overseas qualifications using UCAS’ International Qualifications guide for entry to UK universities in 2015 [22]. Each student’s educational level was then calculated, based upon the highest level of educational qualification that they had obtained prior to entry to university, giving them a score between 1 and 7. Occupational prestige was then calculated for each student’s highest-earning parent, classifying each occupation on Barratt’s 9-point ordinal scale on the basis of approximate similarity to the existing job categories on the scale. Social Status–a proxy for SES–was then calculated by multiplying each student’s score for educational level by 3, each student’s occupational prestige score by 5, and taking the sum of the two (reflecting Barratt’s recommendation of weighting the contributions of these two variables to social status 5:3 in favor of occupational prestige). This gave each student a Social Status score between 8 and 66. Students for whom either qualification data or parental occupation data were missing were coded as missing data on this variable.

As the institution is known to have adopted anonymous marking in the 2005–2006 academic year, a new dichotomous variable was calculated to classify students as either never having had anonymous marking (i.e. those who had left the university prior to the start of the 2005–2006 academic year) or only having anonymous marking (i.e. those who only joined the university after the 2004–2005 academic year). To simplify analyses and accentuate the potential impact that anonymous marking may have had on assessment grades, all students who did not clearly fall into one of these two categories was coded as a missing value on this variable.

### Ethics statement

Ethical approval for this study was gained from the Aston Business School Ethics Committee at Aston University. Students are made aware upon induction that their anonymized data may periodically be used for research purposes.

## Results

### Ethnicity

Descriptive statistics for written examinations, coursework and oral examinations for each of the three ethnic groups examined in the study are shown in Table 1 below. Group mean percentage scores and standard deviations are reported for the overall sample, for those students pre-implementation of anonymous marking and those post-anonymous marking.

**Table 1:** Means (standard deviations) of assessment percentage scores by ethnic origin.

	Pre-implementation			Post-implementation			Difference		
	Exam	CW	Oral	Exam	CW	Oral	Exam	CW	Oral
White	58.7 (10.5)	64.3 (8.9)	65.9 (10.0)	59.8 (12.0)	65.6 (9.1)	68.1 (10.5)	1.1	1.3	2.2
Asian	54.1 (12.0)	61.4 (9.7)	64.2 (10.9)	57.2 (11.3)	63.2 (9.0)	66.0 (11.7)	3.1	1.8	1.8
Black	52.1 (12.9)	60.2 (10.3)	58.9 (16.3)	54.5 (12.4)	63.3 (9.8)	65.6 (13.4)	2.4	3.1	6.7

CW, coursework.

From observation of the figures in Table 1, there appears to have been a slight narrowing of differences in both written examination and coursework marks between the highest scoring group (White students) and the lowest scoring group (Black students), suggesting that implementation of anonymous marking has had the effect of reducing the performance differential between these groups. Furthermore, post-implementation, the magnitude of difference between White students and Black students is larger for both written and oral examinations than it is for coursework, suggesting that Black students may be being disadvantaged by assessment through examinations in spite of anonymous marking.

In order to quantify these differences, Cohen’s *d* was calculated between both of the minority ethnic groups considered in the study and the White majority group. These values are shown in Table 2 below.

**Table 2:** Cohen’s *d* between minority ethnic groups and the White majority.

	Pre-implementation			Post-implementation			Difference		
	Exam	CW	Oral	Exam	CW	Oral	Exam	CW	Oral
Asian	0.4	0.3	0.2	0.2	0.3	0.2	-0.2	0.0	0.0
Black	0.6	0.4	0.5	0.4	0.2	0.2	-0.2	-0.2	-0.3

CW, coursework.

*Note*. A positive value indicates a difference in favor of the White majority. A negative value would indicate a difference in favor of the ethnic group to which it pertains.

Table 2 indicates that there is a trend to indicate that White-Black differences in written and oral examination performance have been substantially reduced post-implementation. Similarly, White-Asian differences have been reduced with the introduction of anonymous marking, though only in the case of written examinations.

To further investigate these patterns, each mean assessment mark was regressed onto the dummy variables representing Asian-non-Asian and Black-non-Black ethnic classifications, both pre- and post-implementation. A summary of the linear regressions conducted is shown in Table 3.

**Table 3:** Regression of assessment marks onto ethnicity.

	B (S.E.)	*β*	*R*^*2*^
*Pre-Implementation*			
Exam			.04
Constant	58.69 (0.16)		
Asian	-4.35 (0.23)	-0.20^a^	
Black	-5.67 (0.61)	-0.10^a^	
Coursework			.02
Constant	64.26 (0.12)		
Asian	-2.17 (0.18)	-0.13^a^	
Black	-3.27 (0.47)	-0.07^a^	
Oral			.01
Constant	66.12 (0.18)		
Asian	-1.79 (0.27)	-0.09^a^	
Black	-4.89 (0.72)	-0.09^a^	
*Post-Implementation*			
Exam			.02
Constant	59.82 (0.14)		
Asian	-2.66 (0.18)	-0.11^a^	
Black	-5.30 (0.33)	-0.12^a^	
Coursework			.02
Constant	65.64 (0.11)		
Asian	-2.48 (0.14)	-0.14^a^	
Black	-2.36 (0.26)	-0.07^a^	
Oral			.01
Constant	68.10 (0.17)		
Asian	-2.13 (0.23)	-0.09^a^	
Black	-2.45 (0.42)	-0.06^a^	

^a^*p* < .001.

From Table 3, there does appear to have been a slight reduction in the variance in assessment scores explained by ethnic origin since implementation of anonymous marking. However, on examination of the changes in B coefficients, this amounts to very small differences in practical terms. For Black students, the mean difference between their marks and those of the White majority on these assessments only appears to have been reduced by approximately two thirds of a percentage point. In the case of Asian students, differences between their marks in written examination marks and those of the White majority have reduced by around 1.5% following implementation of anonymous marking. However, performance differences between Asian and White students in their coursework marks appear to have slightly widened, the B coefficients indicating an increase of around a third of a percentage point post-implementation versus pre-implementation. These findings only partially support *H*_*1*_.

To address *H*_*2*_, it seemed most sensible to consider ethnic differences in assessment performance post-implementation of anonymous marking, to control for any potential effects of assessor bias in written examination and coursework marks. Examining the differences between the variances explained in assessment scores by ethnic group post-implementation, it appears that there is very little difference between assessment methods. The B coefficients indicate that written examinations do favor White students over Black students by around 5%, compared to a difference of only around 2% for coursework, but the difference in oral examination marks is similarly only around 2%. Furthermore, performance differences between Asian and White students appear to be relative consistent across assessment methods, showing assessment score differences of approximately 2% in favor of the White majority. This does not support *H*_*2*_.

### Gender

To investigate group performance differences between male and female students, means and standard deviations of assessment marks were calculated for the overall sample, pre- and post-implementation. These results are shown in Table 4.

**Table 4:** Descriptive statistics of mean assessment mark by gender.

	Pre-implementation			Post-implementation			Difference		
	Exam	CW	Oral	Exam	CW	Oral	Exam	CW	Oral
Female	59.0 (9.7)	64.7 (7.7)	66.2 (8.8)	59.5 (10.9)	64.9 (8.4)	67.8 (10.4)	0.5	0.2	1.6
Male	54.3 (12.6)	61.4 (10.6)	63.5 (12.5)	56.3 (12.6)	63.3 (10.0)	65.8 (12.1)	2.0	1.9	2.3

CW, coursework.

Examining Table 4, there seems to have been a very slight narrowing of gender differences since implementation of anonymous marking. Additionally, there do appear to be larger gender differences post-implementation in exam marks than in coursework, though these differences favor female students. As before, to quantify these differences, Cohen’s *d* was calculated between male and female groups. These values are shown in Table 5 below.

**Table 5:** Cohen’s *d* between male and female groups.

Pre-implementation			Post-implementation			Difference		
Exam	CW	Oral	Exam	CW	Oral	Exam	CW	Oral
0.4	0.4	0.2	0.3	0.2	0.2	-0.1	-0.2	-0.0

CW, coursework.

*Note*. A positive value indicates a difference in favor of females. A negative value would indicate a difference in favor of males.

As was the case for ethnic group differences in the previous analyses, gender-based performance differences appear to have reduced between male and female students with the implementation of anonymous marking, though only in the case of examinations and coursework. Additionally, the magnitude of these reductions seems only to be relatively modest.

To investigate whether these differences are indicative of a true reduction in gender differences, a series of linear regression analyses were conducted, regressing assessment marks onto gender both pre- and post-implementation.

It would appear, from Table 6, that there has been a narrowing of gender differences in written examination and coursework scores since implementation of anonymous marking (variance in assessment scores that can be explained by gender having approximately halved across the board since implementation). However, when examining the change in B coefficients pre- and post-implementation, this only amounts to a mean reduction in marks on these assessments of 0.95 of a percentage point, a very small amount in practical terms. These results only partially support *H*_*1*_.

**Table 6:** Regression of assessment marks onto gender.

	B (S.E.)	*β*	*R*^*2*^
*Pre-Implementation*			
Exam			.03
Constant	58.46 (0.16)		
Gender	-3.92 (0.22)	-0.18^a^	
Coursework			.03
Constant	64.65 (0.12)		
Gender	-2.93 (0.17)	-0.17^a^	
Oral			.01
Constant	66.14 (0.18)		
Gender	-2.18 (0.26)	-.011^a^	
*Post-Implementation*			
Exam			.02
Constant	59.54 (0.12)		
Gender	-3.27 (0.17)	-0.14^a^	
Coursework			.01
Constant	64.94 (-1.68)		
Gender	-1.68 (0.13)	-0.09^a^	
Oral			.01
Constant	67.80 (0.15)		
Gender	-2.00 (0.21)	-0.09^a^	

^a^*p* < .001.

To address *H*_*2*,_ the differences in variance explained post-implementation between examination marks and coursework marks were examined. Gender does explain less of the variance in coursework marks than it does in examination marks, though this is once again relatively small in practical terms. The B coefficients suggest gender differences in written examination scores of 3.27% and in oral examination scores of 2%. When compared to the gender difference in coursework scores of 1.68%, this demonstrates that the real-world differences between these modes of assessment is only slight. Furthermore, all performance differences were found to be in favor of females, so these findings do not support *H*_*2*_.

### Socio-economic status

Two conceptualizations of Socio-economic Status were included in the analyses. Firstly, means and standard deviations of assessment marks for students from non-selective and selective schools were calculated for the overall sample. These results are shown in Table 7. As it was not expected that anonymous marking would have had any impact on socio-economic group differences, these results are omitted from the table.

**Table 7:** Descriptive statistics for assessment mark by school type.

	Overall		
	Exam	Coursework	Oral
Non-selective	55.5 (11.4)	63.6 (9.2)	66.1 (10.8)
Selective	56.3 (10.3)	63.8 (8.9)	65.9 (10.5)

Across all three modes of assessment, there was very little difference in the mean assessment scores between students from selective and non-selective schools. Linear regression analyses were then conducted, regressing assessment marks onto school type.

When each assessment score variable was regressed onto school type (shown in Table 8), the model in each case explained less than 1% of the variance in mean assessment scores. In practical terms, by examining the B coefficients, school type accounts for a difference of less than one percentage point in assessment scores across the board.

**Table 8:** Regression of assessment marks onto school type.

	B (S.E.)	*β*	*R*^*2*^
Exam			.00
Constant	55.53 (0.11)		
School Type	0.77 (0.22)	0.03^a^	
Coursework			.00
Constant	63.65 (0.09)		
School Type	0.17 (0.18)	0.01	
Oral			.00
Constant	66.07 (0.12)		
School Type	-0.13 (0.23)	-0.01	

^a^*p* < .001.

As Social Status, as represented by the BSMSS, is not categorical in nature, it does not lend itself well to similar comparison. Instead, assessment marks were regressed on to Social Status score. The results of this analysis are shown in Table 9.

**Table 9:** Regression of assessment marks onto Social Status score.

	B (S.E.)	*β*	*R*^*2*^
Exam			.01
Constant	52.94 (0.36)		
Social Status	0.08 (0.01)	0.09^a^	
Coursework			.00
Constant	63.50 (0.30)		
Social Status	0.02 (0.01)	0.02^b^	
Oral			.00
Constant	65.11 (0.39)		
Social Status	0.03 (0.01)	0.03^c^	

^a^*p* < .001.

^b^*p* < .05.

^c^*p* < .01.

As in the previous analysis, the measure of social status used explained very little variance in marks for any of the assessment methods examined. When considered alongside the findings from the previous analysis, these findings do not support *H*_*3*_.

## Discussion

When considered in their totality, the findings of the present research are somewhat surprising. Despite the supporters of anonymous marking claiming that its implementation has led to fairer assessment in Higher Education [11], the present study suggests that anonymous marking initiatives–at least in the present case–have done little to eliminate between-group mean performance differences. Ethnic, gender and socio-environmental differences seem to be pervasive in academia, even after interventions aimed to reduce them.

However, when interpreted in the context of practical impact, these findings paint a rather more optimistic picture: Although these differences do exist, practically they are very small at the group level. The largest observed differences in explained variance–those between White and Black students–only amounts to a difference between these groups of, at most, 5% in terms of their actual examination performance. While even a single percentage point can potentially represent the difference between grades–or even, in some cases, the difference between degree classifications, it is the opinion of the author that this does not support the assertion that these assessment methods show evidence of bias favoring one group over another.

Examining this assertion more fully, there is little evidence to support the hypothesis that certain ethnic groups suffer the ill effects of Stereotype Threat in examinations to which Steele & Aronson [16] proposed they were subject in cognitive tests, and no evidence whatsoever to support that this effect is present for females. Furthermore, the variance in written examination and coursework marks has reduced since implementation of anonymous marking. However, a similar pattern was observed in almost all cases for the variance that could be explained in oral examination marks. Clearly, oral examinations cannot have benefitted from anonymous marking, yet the same pattern of reduction in performance differences has been observed. There are several possible explanations for this observation. Firstly, one explanation could be in terms of the quality of teaching practices that have evolved in the institution over the last twelve years. It is possible that better teaching has led to students learning–and being able to demonstrate their learning–at a more consistent level across the teaching body. Secondly, it is entirely possible that the implementation of anonymous marking has had the knock-on effect of making assessors more aware of their own potential for unconscious bias, which has led them to assess students more fairly in all modes of assessment. Finally, the implementation of anonymous marking may have demonstrated to students that the university has made a commitment to the ethical treatment to those in all social groups in their assessment processes. The students’ resultant perception of procedural justice (a facet of organizational justice) may have served to increase their performance across all modes of assessment, in much the same way as it has been observed to increase job performance [23].

### Recommendations for practice

On the basis of these findings, a number of recommendations can be made for both practitioners and researchers in Higher Education. The key recommendation from the study is that further research be targeted towards the examination of the impact of anonymous marking on performance differences. It would be reactionary for the authors to recommend, based on the present study’s findings alone, that anonymous marking be revoked in institutions in which it has been implemented, or not to implement it in institutions that have not already done so. Rather, anonymous marking’s impact on student performance–both directly and indirectly–needs to be considered carefully to establish whether or not it is functioning in the way in which we assume it does. Since anonymous marking is a potentially costly practice for institutions to implement, training to increase assessors’ awareness of their own potential unconscious biases might prove to be a more cost-effective option for ameliorating performance differences.

The secondary recommendation for practitioners would be to recognize that written examinations do not disadvantage certain social groups as much as one might have been led to believe. One approach to addressing the performance differences in written examinations that is proving increasingly popular in Higher Education practice in recent years is the implementation of modules that do not use written examinations to assess students. Proponents of this approach claim that this provides fair assessment for more students than the classic assessment mode of midterm paper + final written examination [1]. However, the present findings would suggest that any increase in the fairness of assessment by implementation of such measures is borderline at best, and at worst exacerbates the potential for–and incidence of–plagiarism and collusion [2]. Practitioners should, therefore, reconsider their position on all-coursework modules on the basis of these findings, at least until further research has been conducted.

### Limitations and recommendations for future research

The limitations of the present study are focused on the data that is contained within the obtained archive. In particular, the data used to construct the measure of Social Status based on the work of Barratt [18] only allowed for a partial representation of the construct, a proxy for social status (it being itself a proxy for SES). Barratt maintained that, for students in full-time education, personal educational level should be discounted, the educational component of a student’s social status score being derived exclusively from that of their parent(s) or guardian(s). Unfortunately, this data is not recorded by the institution upon commencement–or completion–of their degree program, making the calculation of social status scores as Barratt intended impossible.

As discussed above, future research needs to focus on how it might be that anonymous marking might have led to performance increases in HE assessment across the board, not just in written assessments. A wider point to consider is to do with the way in which we, as academics, assume that these performance differences come about. Instead of focusing on differences in academic results between groups as being an artefact of idiosyncratic rating tendencies within the assessor, future research in this area should seek to understand these differences in terms of the *mechanisms* by which performance differences manifest at the group level. This is, to date, something that has been frequently neglected in the educational assessment literature. By contrast, the field of occupational psychology has devoted much attention to the study of the factors that influence performance, albeit in the context of job performance. In addition to the research described above on the link between perceived procedural justice and performance, a wealth of research exists on its antecedents. A commonly-accepted model of job performance proposes that differences in job performance come about through the interaction of three distinct variables, job knowledge, skills / work habits, and motivation [24], a fourth antecedent in personality traits having been proposed as a revision to the existing model [25].

Using job performance as an analogue of academic performance (at both the macroscopic, degree level and the microscopic, assessment level), performance differences between groups in higher education assessment can be understood in terms of the interaction between a number of factors. Future research into performance differences in higher education assessment should, at the very least, take steps to understand group performance differences in the context of these influencing factors, though the authors recognize that it is far from easy in practical terms to obtain these kinds of data in large quantities.

## Supporting information

S1 File Data file. (SAV)
